# Arc of Buhler pseudoaneurysm complicated by an arterioportal fistula: a combined endovascular and open surgical approach

**DOI:** 10.1093/jscr/rjag645

**Published:** 2026-08-25

**Authors:** Islam Elhelf, Kate Elmore, Shaan Haider, Sarah Hanna, Abul Rahman Abualruz, Lane Estes, Steven Colquhoun

**Affiliations:** Department of Radiology, Medical College of Georgia, 1120 15th Street, Augusta, GA 30912, United States; Department of Radiology, Emory University School of Medicine, 201 Dowman Dr., Atlanta, GA 30222, United States; Medical College of Georgia, Class of 2028, 1120 15th Street, Augusta, GA 30912, United States; Medical College of Georgia, 1120 15th Street, Augusta, GA 30912, United States; Keck School of Medicine, University of Southern California, 1975 Zonal Ave., Los Angeles, CA 90033, United States; Department of Radiology, Medical College of Georgia, 1120 15th Street, Augusta, GA 30912, United States; Department of Surgery, Medical College of Georgia, 1120 15th Street, Augusta, GA 30912, United States

**Keywords:** Arc of Buhler, pseudoaneurysm, arterioportal fistula, visceral artery aneurysm

## Abstract

The Arc of Buhler (AoB) is a rare anatomic variant characterized by direct anastomosis between the celiac artery and the superior mesenteric artery. There is a relative paucity of data regarding this anatomy, likely due to its rather low incidence. The most common pathology associated with AoB is aneurysm formation, and while AoB aneurysm is exceedingly rare, a pseudoaneurysm appears even more so. We present a case of an AoB pseudoaneurysm in a 62-year-old male which was complicated by an arterioportal fistula (APF) between the pseudoaneurysm and the main portal vein. A multidisciplinary team of surgeons, interventional radiologists, and body radiologists worked together to treat this complex pathology. To our knowledge, this is the first reported case of an AoB pseudoaneurysm with an APF. This case emphasizes the importance of multispecialty collaboration in treating such unusual and complex vascular pathology.

## Introduction

First described in 1904, the Arc of Buhler (AoB) is a rare direct anatomic connection between the celiac artery and the superior mesenteric artery [1]. Some theories propose this is a failure of regression of the ventral anastomosis of the 10th and 13th segmental arteries of the aorta formed in utero [2]. The most common pathology associated with AoB is aneurysm formation, which is almost always symptomatic [3].

There is a relative paucity of data regarding the AoB, likely due to its low incidence. A recent meta-analysis by Kowalczyk *et al.* determined that the pooled prevalence of AoB in the general population is ~1.7% [3]. The overall incidence of visceral artery aneurysms is estimated to be 0.1%–2%, with 5% of these being pseudoaneurysms [4]. Therefore, an AoB aneurysm appears to be exceedingly rare, with a pseudoaneurysm becoming even more uncommon [3, 4].

Here we present a case of an AoB pseudoaneurysm (PSA) complicated by an arterioportal fistula (APF) to the main portal vein.

## Case report

**Figure 1:**
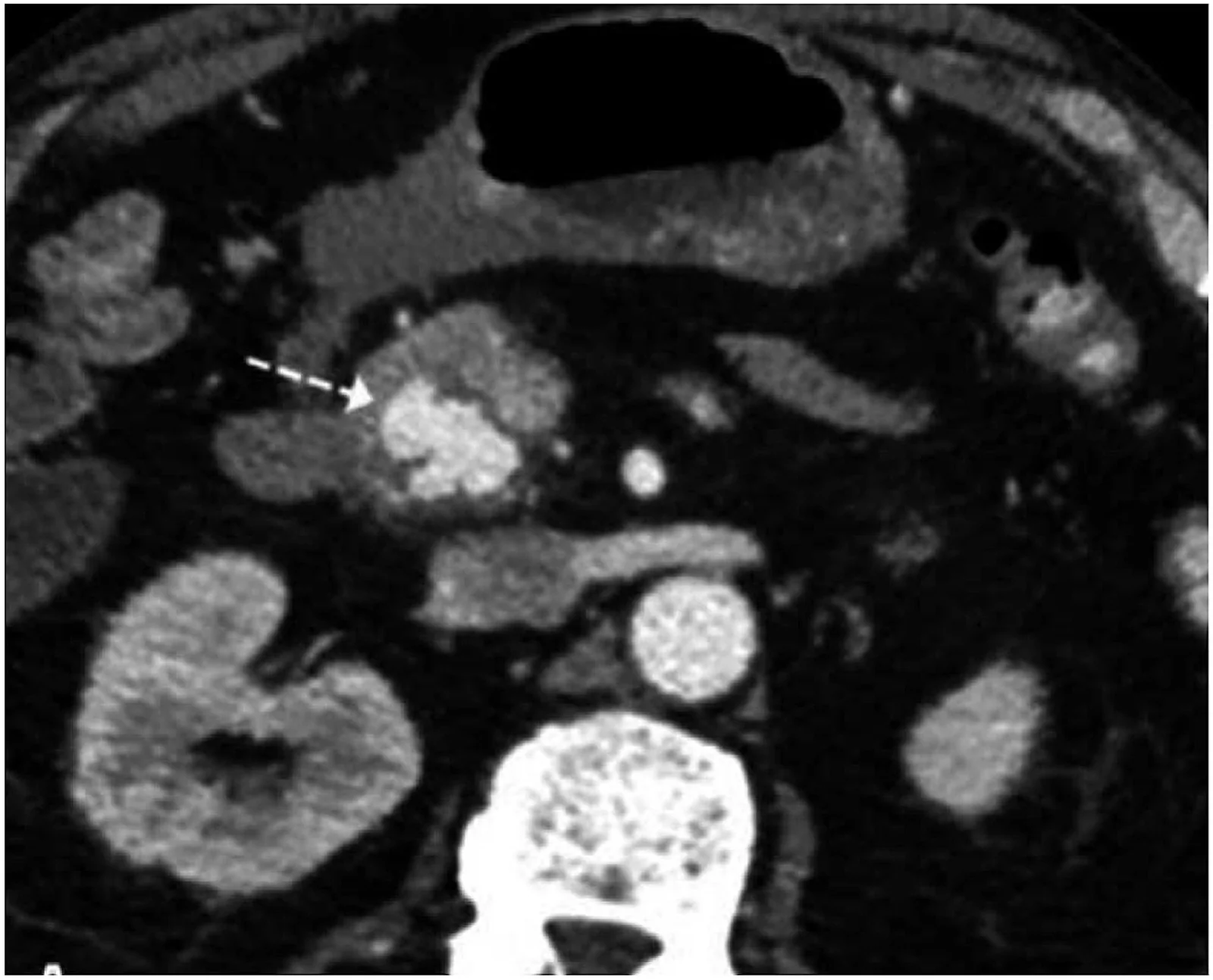
Axial contrast-enhanced CT through the abdomen at the level of the pancreatic head with arrows pointing to a large pseudoaneurysm.

The patient is a 62-year-old male with hypertension, diabetes, and alcohol-induced chronic pancreatitis who presented with severe abdominal pain. A contrast-enhanced computed tomography (CT) revealed changes of acute-on-chronic pancreatitis associated with a large walled-off necrotic collection at the region of the pancreatic body and tail. There was an incidental note of an irregularly shaped, avidly enhancing lesion at the region of the pancreatic head measuring ~2.5 × 1.9 cm. This lesion appeared intimately associated with an anomalous and torturous artery in region of the pancreatic head that appeared to communicate with the superior mesenteric artery (SMA). Additionally, early opacification of the portal vein was also appreciated, raising concern for fistulous communication with this vascular lesion (Fig. 1). A differential diagnosis was provided that included an arteriovenous malformation versus an APF. Management included opinions from both interventional radiologists (IR) and surgeons.

**Figure 2:**
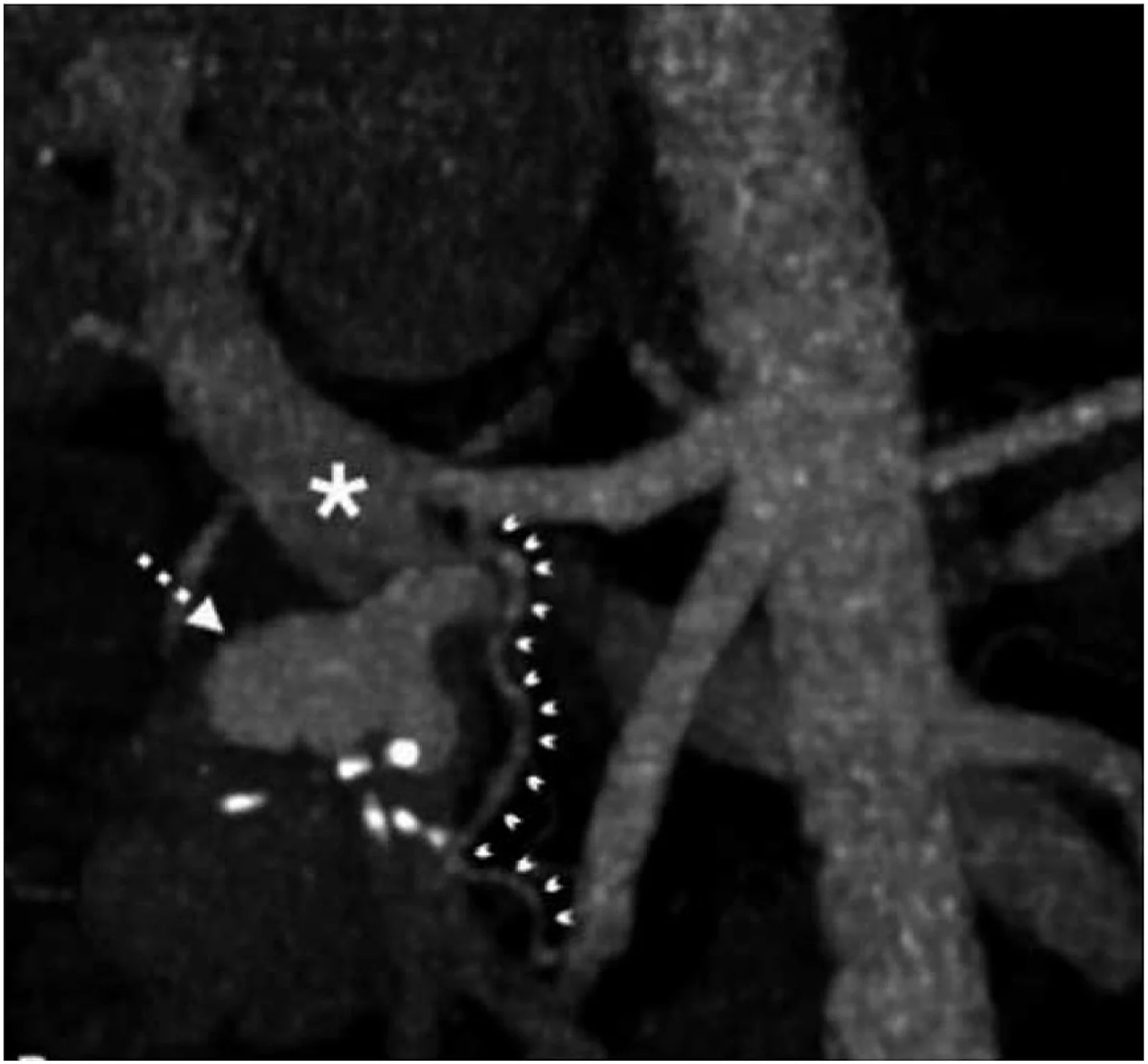
Maximum-intensity-projection reformat, orthogonal to the Arc of Buhler (arrowheads), showing both the pseudoaneurysm (dashed arrow) and the early filling portal vein (asterisk), in keeping with APF.

**Figure 3:**
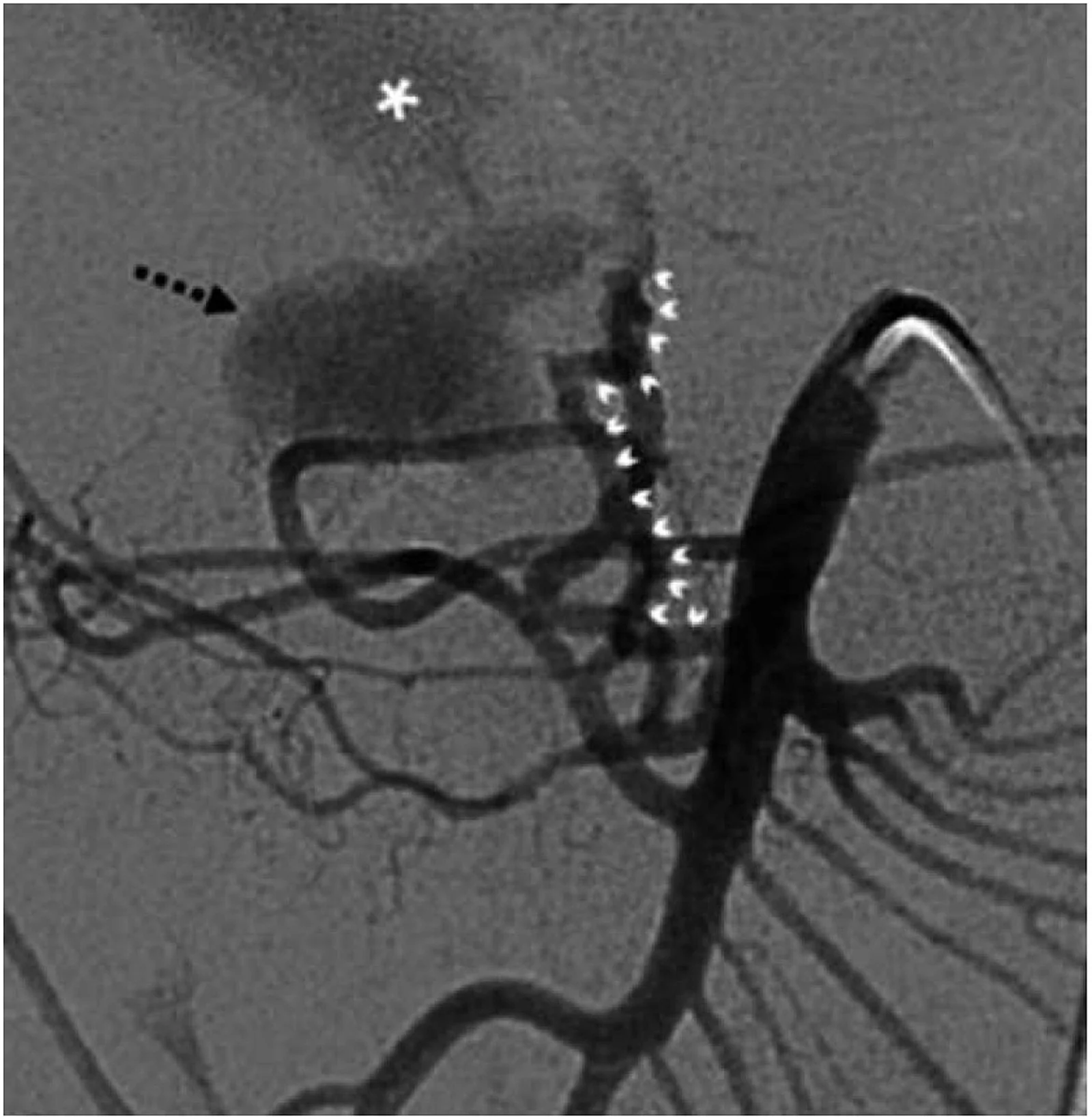
AP view of superior mesenteric angiogram showing the AoB (arrowheads), PSA originating from the AoB (arrow) and early filling of the portal vein through an APF (asterisk).

An angiogram revealed an irregularly shaped PSA communicating with a hypertrophied AoB, demonstrating high-flow fistulous outflow into the portal vein at the confluence of the splenic and superior mesenteric veins (Figs 2 and 3). Several attempts to access the AoB from the common hepatic artery (CHA) side were unsuccessful due to the acute angle of origin of the AoB from the CHA.

**Figure 4:**
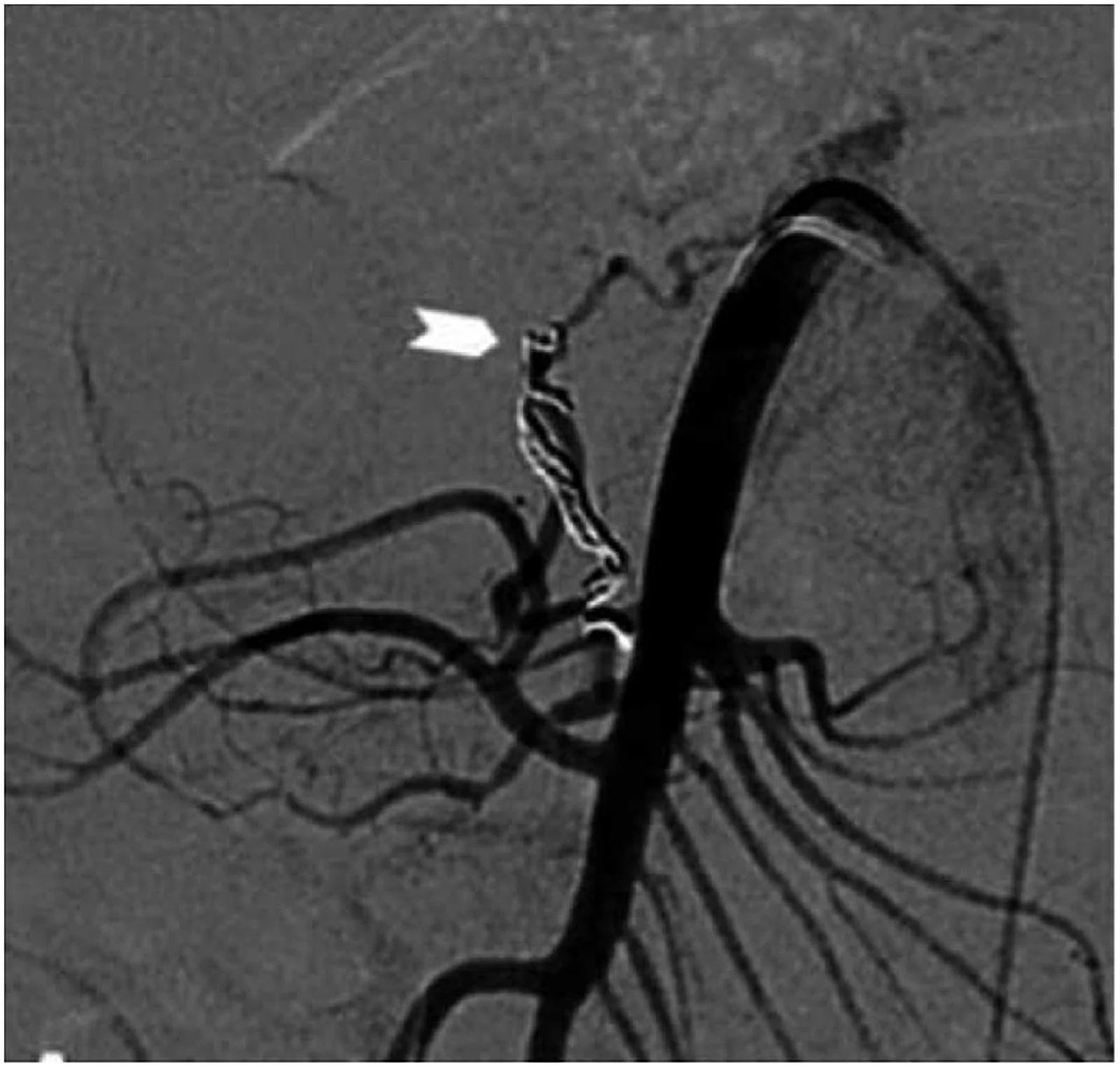
Angiogram from superior mesenteric artery after coil embolization of the AoB showing no evidence of PSA filling from SMA side (arrowhead pointing to distal end of coil at the level of the neck of the PSA). Note there is a separate artery projecting beyond the coil.

**Figure 5:**
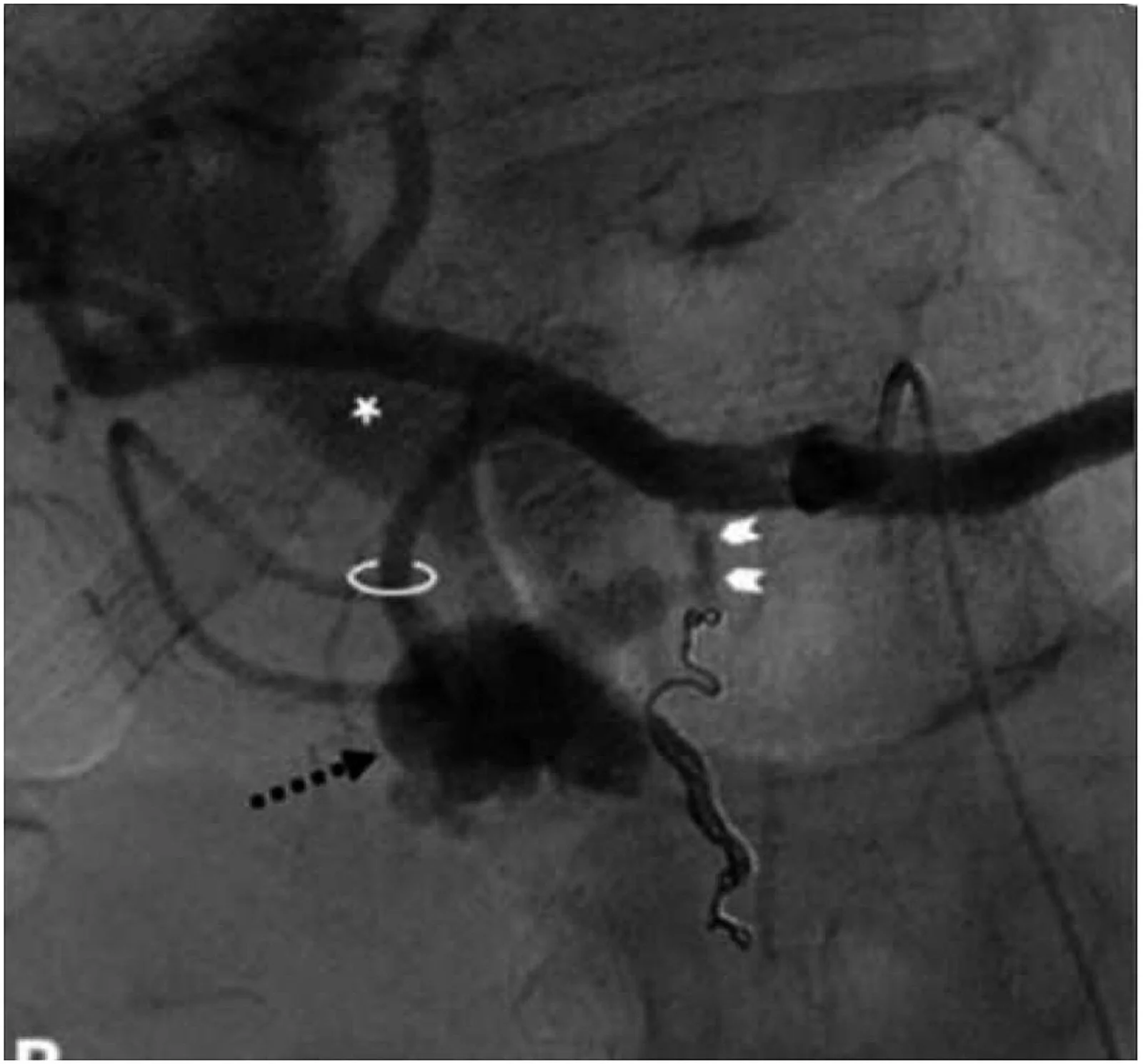
Angiogram from celiac trunk after AoB coiling from the SMA side showing progressive slow filling of PSA (dashed arrow) from the common hepatic artery side through the proximal segment of the AoB (arrowheads). There is a persistent APF with opacification of the portal vein (asterisk). The gastroduodenal artery is circled as an anatomic landmark.

The decision was made to access the AoB from the SMA side, and after several attempts it was successfully embolized with coils. However, the final angiogram showed persistent supply of the PSA from the CHA side of the arc, but with slowed shunting through the APF (Figs 4 and 5).

A repeat CT was performed 5 days after coil embolization, which showed a slight decrease in size of the PSA, now measuring 2.2 × 1.7 cm (previously 2.5 × 1.9 cm) with no significant flow through the embolized SMA contribution to the AoB. However, there was persistent opacification of the PSA from the CHA side with persistent APF. At this point, the decision was made to proceed with surgery to control the vascular contribution from the CHA side. Surgery identified two branch arteries arising from the CHA proximal to the origin of the gastroduodenal artery. The larger of these two branches was felt to be supplying the PSA and was clipped.

**Figure 6:**
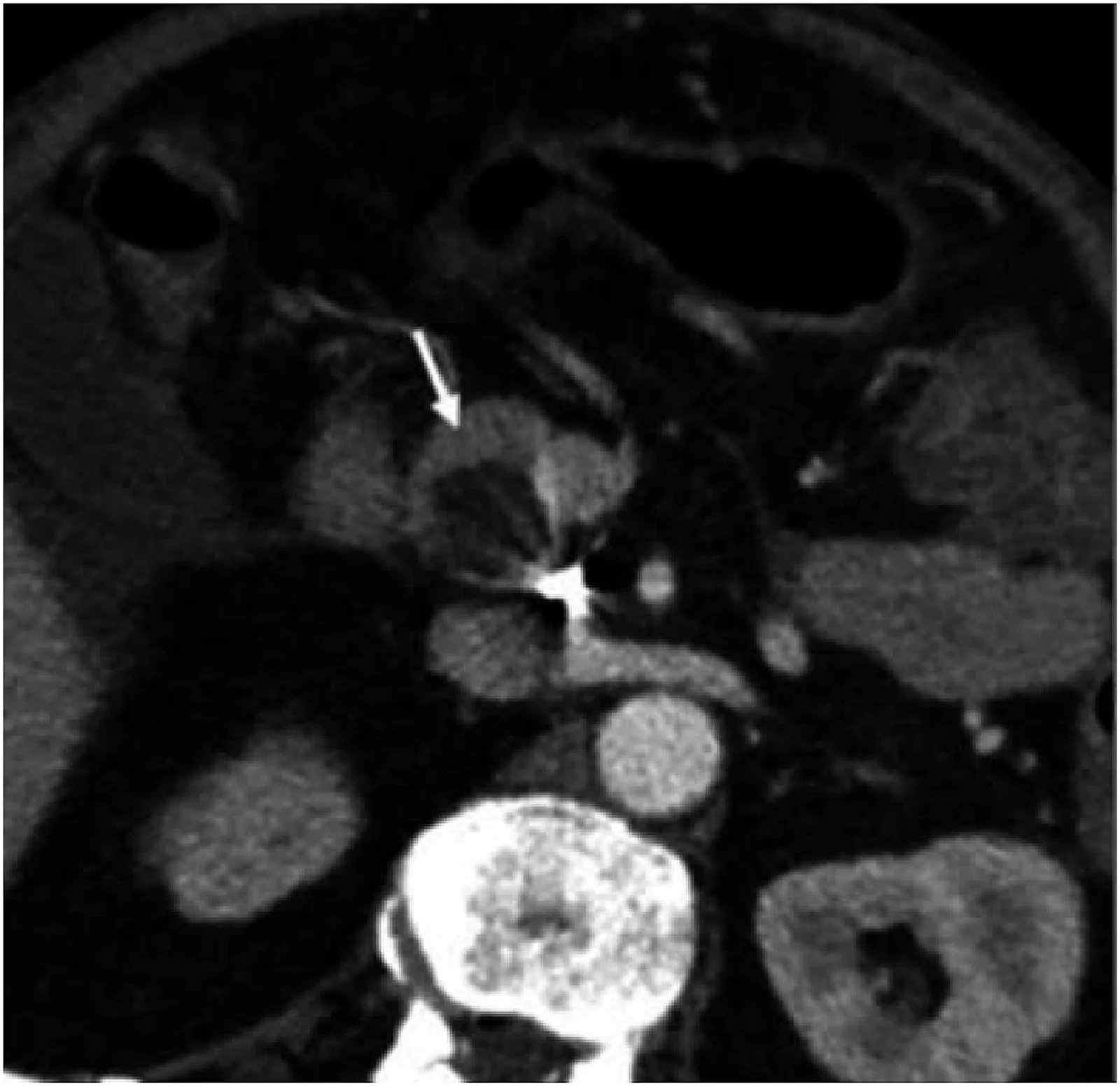
Follow up contrast enhanced CT scan showing complete thrombosis of the PSA.

Another CT was performed which showed no change in size of PSA. To assess post-surgical vascular changes, angiography was performed and showed a persistent feeding artery to the PSA form the CHA side, but this was again unable to accessed endovascularly. The next day, the patient returned to the OR for repeat ligation. The relevant feeding artery from the CHA side was identified with intraoperative ultrasound and ligated. Repeat CT was done and showed significant reduction of the PSA with peripheral mural thrombus formation with the aneurysm, now measuring 1.9 × 1.5 cm. The patient continued to receive treatment for necrotizing pancreatitis and was eventually discharged home in stable condition. Follow-up imaging performed 43 days after the last CT showed complete thrombosis of the PSA and resolution of the APF (Fig. 6). There was no subsequent portal vein thrombosis; patient never received antiplatelets/anticoagulation and d-dimer levels were not assessed.

## Discussion

The initial approach to treating AoB aneurysms is based on the standard of care for visceral and mesenteric aneurysms and PSAs elsewhere: an endovascular approach given the high rate of PSA rupture and subsequent mortality [5]. The endovascular approach may confer a higher success rate and a lower complication rate in treatment of PSAs especially, as these often arise in the setting of inflammation and injury which would complicate open repair [5]. However, complex PSAs may pose challanges such as acute angles preventing guidewire access, imminent risk of portal venous thrombosis or PSA rupture – factors that all necessitate consideration for open surgery.

In this patient’s case, endovascularly coiling the main AoB supply from the SMA was important to decrease the extent of high flow AP shunting, allowing for partial thrombosis of the PSA. However, failure to completely embolize the PSA during the initial session and persistent AP shunting highlights the importance of isolating all potential collateral branches supplying the PSA. An open surgical approach using intraoperative Doppler ultrasound to identify the source of residual arterial inflow allowed for successful ligation of the contributing artery. This resulted in a significant reduction of AP fistulous flow that allowed for progressive thrombosis of the PSA and eventual resolution of the fistula.

Alternative treatment approaches such as direct thrombin injection into the PSA were not deemed appropriate as it carried a higher risk of thrombin migration and subsequent portal venous thrombosis.

AoB aneurysms are relatively rare with a total of 12 cases in the literature (Table 1). However, to our knowledge this is the first case associated with APF. This high flow, fistulous communication significantly increased the complexity of treating the PSA and brought a unique opportunity for collaboration between surgery and IR.

**Table 1:** Published cases of Arc of Buhler aneurysms.

Author	Pseudoaneurysm or true aneurysm and its integrity	Treatment	Outcome
Ehemann *et al*. [4]	Pseudoaneurysm; ruptured	Endovascular embolization of the AoB and aneurysm sac	Success
Dubel *et al*. [5]	Not specified; intact	Endovascular embolization of the AoB and aneurysm sac	Success
Kugai *et al*. [6]	Not specified; intact	2 failed endovascular embolization attempts, successful surgical aneurysmectomy	Success
Myers *et al*. [7]	True aneurysm; intact	Surgical aneurysmectomy	Success
Jeong *et al*. [8]	Not specified; intact	Endovascular embolization of the AoB and aneurysm sac	Success
Jayia *et al*. [9]	Not specified; intact	Endovascular embolization, not otherwise specified	Success
Sugihara *et al*. [10]	Not specified; intact	Endovascular embolization of aneurysm sac	Success
Mohapatra *et al*. [11]	Not specified; ruptured	Endovascular embolization, not otherwise specified	Success
Ong *et al*. [12]	True aneurysm; ruptured	Endovascular embolization of the entire AoB	Success
Biswas *et al*. [13]	Pseudoaneurysm; initially intact then ruptured	Observation while intact, then surgery once ruptured	Death
Abe *et al*. [14]	Not specified; ruptured	Endovascular embolization, not otherwise specified	Success
Quaretti *et al*. [15]	Not specified, intact	Endovascular embolization of aneurysm sac with stent grafting of the SMA	Success

## References

[1] Buhler A . Uber eine anastomose zwischen den stammen der art. Coeliaca und art. Mesenterica superior. Morphol Jahrb 1904;32:185–8.

[2] Tandler J . Über die Varietäten der arteria coeliaca und derez Entwickelung. Anatomische Hefte 1904;25:473–500.

[3] Kowalczyk KA, Pękala JR, Kawzowicz M et al. The meta-analysis and systematic review of prevalence and clinical anatomy of the arc of Buhler. Sci Rep 2023;13:9183. 10.1038/s41598-023-36316-9 PMC10244378 37280432

[4] Ehemann J, Kim J. Rare vascular complication of ESWL pseudoaneurysm of arc of Buhler. BMJ Case Reports 2023;16:e256089. 10.1136/bcr-2023-256089 PMC10373728 37491127

[5] Dubel G, Ahn S, Saeed M. Interventional management of arc of Buhler aneurysm. Semin Intervent Radiol 2007;24:076–81. 10.1055/s-2007-971193 PMC3036356 21326742

[6] Kugai T, Nakamoto T, Tamaki N et al. Aneurysm of an artery of Buhler: an unusual case. J Vasc Surg 1996;23:537–8. 10.1016/s0741-5214(96)80024-3 8601901

[7] Myers JL, Losseff SV, Gomes MN. Surgical repair of an aneurysm of the arc of Buhler in a patient with von Recklinghausen’s disease. Eur J Vasc Endovasc Surg 1998;16:540–2. 10.1016/s1078-5884(98)80249-9 9894498

[8] Jeong SJ, Lim NY, Jang NK et al. Transcatheter coil embolization of an arc of Buhler aneurysm. Korean J Radiol 2008;9:S77–80. 10.3348/kjr.2008.9.s.s77 18607134 PMC2627200

[9] Jayia P, Hosney S, Subramanian A et al. Arc of Buhler aneurysm: a rare cause of obstructive jaundice. Vasc Endovascular Surg 2011;45:92. 10.1177/1538574410379652 21075755

[10] Sugihara F, Murata S, Uchiyama F et al. Successful coil embolization of an aneurysm in the arc of Bühler. J Nippon Med Sch 2016;83:196–8. 10.1272/jnms.83.196 27890893

[11] Mohapatra S, Moradi D, Broder A. A rare cause of post-sphincterotomy bleeding. Gastroenterology 2017;153:1193–4. 10.1053/j.gastro.2017.04.007 28987754

[12] Ong DY, Pua U. Coil embolization of arc of Buhler aneurysm rupture. Case Rep Radiol 2020;2020:1–3. 10.1155/2020/8855946 PMC7748894 33381343

[13] Biswas S, Gogna S. Arc of Buhler pseudoaneurysm causing fatal retroperitoneal hemorrhage; a rare case report and discussion of relevant literature. Bull Emerg Trauma 2019;7:183–6. 10.29252/beat-070215 31198809 PMC6555205

[14] Abe K, Iijima M, Tominaga K et al. Retroperitoneal hematoma: rupture of aneurysm in the arc of Bühler caused by median arcuate ligament syndrome. Clin Med Insights Case Rep 2019;12: 117954761982871. 10.1177/1179547619828716 PMC6376540 30792583

[15] Quaretti P, Corti R, D’Agostino AM et al. Covered stent assisted coil embolization of large Buhler aneurysm in setting of chronic celiac trunk occlusion. CVIR Endovasc 2024;7:9. 10.1186/s42155-023-00416-4 PMC10781915 38198119

